# Inappropriate antibiotic prescribing for acute respiratory illnesses in outpatient settings in New York City, 2019–2022

**DOI:** 10.1017/ash.2026.10351

**Published:** 2026-04-20

**Authors:** Celina N. Santiago, Katelynn Devinney, Molly M. Kratz, Elise Mantell, Elizabeth Cave, Karen A. Alroy, William G. Greendyke, Nicole Burton

**Affiliations:** Bureau of Communicable Diseases, New York City Department of Health and Mental Hygiene, USA

## Abstract

**Background:**

Inappropriate antibiotic prescribing contributes to antibiotic resistance threats. In outpatient settings, antibiotics are often incorrectly prescribed for acute respiratory illnesses (ARI). Characteristics associated with inappropriate antibiotic prescribing at New York City (NYC) outpatient ARI visits were assessed to identify opportunities for interventions.

**Methods:**

Using IQVIA’s commercial Medical Claims and Longitudinal Prescription datasets, medical diagnosis codes identified outpatient visits for ARI during 2019–2022, which were linked to antibiotics obtained at a pharmacy (as a proxy for prescribed antibiotics) within 3 days post-visit. Univariate analyses were conducted describing visit, patient, and provider characteristics. Modified Poisson regression with robust error variance was used to calculate unadjusted relative risks (RR) and 95% CI for visit-level characteristics associated with inappropriate prescribing.

**Results:**

Among 3,493,444 ARI outpatient visits, 5.1% linked to an inappropriate antibiotic prescription. Among all ARI, bronchitis/bronchiolitis had the highest percentage (25.5% of bronchitis/bronchiolitis visits) and highest risk of inappropriate prescribing relative to asthma/allergy (RR: 18.03; 95% CI: 17.70, 18.38). Adults aged 65–79 years were over twice as likely to be prescribed inappropriate antibiotics relative to children (RR: 2.21; 95% CI: 2.17, 2.25). Inappropriate prescribing was highest in urgent care (8.4%) (RR: 1.25; 95% CI: 1.23, 1.27) relative to offices and among internal medicine physicians (8.0%); relative to these physicians, risk of inappropriate prescribing among all other physician specialties was lower.

**Conclusions:**

Inappropriate antibiotic prescribing at ARI outpatient visits was uncommon. Tailoring interventions to providers such as internal medicine physicians or those in urgent care settings may improve prescribing practices.

## Background

Inappropriate antibiotic prescribing contributes to bacterial resistance to antibiotics. This may include prescribing an antibiotic that is ineffective, for the wrong duration, with a broader spectrum than necessary, or for inappropriate conditions, such as viral infections.^1–3^ Inappropriate prescribing is an urgent public health problem as it also increases health risks to patients and economic costs to health systems.^4,5^

People are often inappropriately prescribed antibiotics for antibiotic-inappropriate acute respiratory illnesses (ARI) (eg, asthma, influenza, and bronchitis) because although their etiologies are viral or non-infectious, their symptoms overlap with those of bacterial infections.^1,6–11^ Assessing prescribing patterns for ARI can guide interventions by highlighting setting types, patient populations, or provider specialties where it occurs frequently.^7,12^ Because most antibiotic prescribing occurs in outpatient settings, where ARI infections are frequently diagnosed, antibiotic stewardship (AS) interventions targeting inappropriate prescribing in these locations may be particularly fruitful.^1,3,12,13^

Medical and prescription claims data from commercial entities can be used to analyze widespread prescribing practices.^9,14–16^ While high volume antibiotic prescribing is used as a proxy for inappropriate antibiotic prescribing, patient diagnosis is necessary to account for the appropriateness of the prescription.^17–19^ To guide AS interventions and research, the aim of this study was to identify visit, patient, and provider characteristics associated with antibiotic prescriptions for antibiotic-inappropriate ARI diagnoses in New York City (NYC) outpatient settings during 2019–2022.

## Methods

### Data sources

This secondary analysis used two commercially available datasets from IQVIA Government Solutions (IQVIA): Medical Claims (Dx) and Longitudinal Prescription Claims (LRx).^15^ These datasets were linked to describe visits where ARI were diagnosed, and an antibiotic was dispensed. IQVIA data come from insurance claims and capture medical claims from around 96% of physicians who are registered with the American Medical Association and between 74 and 94% of mail-order and retail prescriptions. Both datasets are validated by IQVIA to ensure quality and have been previously used by researchers in private and government settings (IQVIA, Falls Church, VA).

The Dx dataset included *International Classification of Diseases, Tenth Revision, Clinical Modification* (*ICD-10-CM*) diagnosis codes that providers designate at each encounter. From this dataset, visits where ARI diagnoses were made for which prescribing an antibiotic would be inappropriate were identified, using a previously established, tiered approach, for patients seen by NYC-based outpatient providers during January 1, 2019–December 31, 2022.^9,20^ Supplemental Table 1 lists the antibiotic-inappropriate *ICD-10-CM* ARI diagnosis codes requested from IQVIA. Outpatient encounters where an antibiotic-inappropriate ARI was diagnosed alongside any diagnoses where antibiotics were always or sometimes appropriate were excluded. Outpatient providers were considered NYC-based according to their mailing address ZIP code. Supplemental Table 2 lists outpatient facility types identified in Dx. Multiple visits by the same patient, to the same provider, on the same day, were defined as a single visit.

Only providers with an individual National Provider Identification Number designation were retained in the analytic dataset. From those, all visits from providers with <10 antibiotic-inappropriate ARI visits, as well as those identified as extreme outliers during the study period, were excluded for stability^9^ and to improve validity, respectively (Supplemental Figure 1).

The LRx dataset included claims for prescriptions obtained from pharmacies, which were used as a proxy for antibiotics prescribed. Prescriptions were deduplicated based on various variables: patient and provider identification numbers, date antibiotic dispensed, prescription type, antibiotic name, dosage form, antibiotic strength, dispensed quantity, and number of days’ supply. An inexact match was used to link visits for ARI to antibiotic prescriptions made within 3 days post-visit using IQVIA’s patient and provider identifiers (sample code provided in Supplemental Material 1). Patient and provider identifiers are persistent de-identified identification numbers generated through IQVIA’s proprietary de-identification algorithm and were used to link the datasets (IQVIA, Falls Church, VA). If more than one antibiotic linked to a visit, it was labeled as multiple, but visits were only dichotomously labeled as having linked to at least one antibiotic or none. All antibiotic prescriptions linking to a patient visit were designated as inappropriate since all diagnoses made were for antibiotic-inappropriate ARI. Only new (ie, non-refill), oral antibiotic prescriptions were included in the analysis. Supplemental Table 3 lists antibiotics identified in the dataset.

### Measures and statistical analysis

Univariate analyses were conducted to assess visit, patient, and provider characteristics. Since during the study period patients could have multiple visits for ARI, providers could see multiple patients for ARI, and patients may see the same provider more than once, the assumption of independence was violated and modified Poisson regression with robust standard error variance was used to calculate unadjusted relative risks (RR) and 95% CIs for the outcome of at least one inappropriate antibiotic prescription received following an outpatient ARI visit.^21^ Example code is provided in Supplemental Material 1. Analyses were completed using SAS Enterprise Guide, version 8.3 (SAS Institute, Cary, NC).

### Ethics

This analysis was given a non-human subjects research determination by the NYC Health Department Institutional Review Board.

## Results

Among 3,493,444 ARI outpatient visits assessed during 2019–2022, 1,765,994 unique patients were seen by 15,143 unique providers. Patient age ranged from 0 to greater than 85 years (age above 85 was suppressed), with 22.7% of visits for pediatric patients ages 0–17 (Table 1). Over half of the visits (58.8%) were for female patients. Most visits (79.2%) were paid for with third party insurance; Medicare and Medicaid represented nearly all remaining visits (12.9% and 7.7% respectively). Approximately half of the visits (55.8%) occurred in an office. Other settings included urgent care (7.9%) and telehealth (6.3%). Most patients had multiple antibiotic-inappropriate ARI visits, with less than one quarter having just one (23.1%) during the four-year period. The most common borough for visits was Manhattan (32.7%). Brooklyn was the most common borough of patient residence (27.8%). Staten Island had the lowest percentage of visits (5.6%); this was also the least common borough of residence (5.6%) (Table 1).

**Table 1.** Outpatient visits^1^ to New York City (NYC) providers for acute respiratory illnesses, 2019–2022

	n (Total N = 3,493,444)	%
**Year of visit**		
2019	939,921	26.9%
2020	801,656	22.9%
2021	805,155	23.0%
2022	946,712	27.1%
**Patient age at visit (years)**		
0–17	791,981	22.7%
18–24	175,654	5.0%
25–44	748,964	21.4%
45–64	974,773	27.9%
65–79	554,475	15.9%
80+	235,552	6.7%
Unknown	12,045	0.3%
**Patient gender^2^**		
Male	1,427,035	40.8%
Female	2,055,550	58.8%
Unknown	10,859	0.3%
**Visit payment type^3^**		
Third Party	2,768,542	79.2%
Medicare (including Part D)	450,253	12.9%
Medicaid	268,604	7.7%
Cash	1	0.0%
Multiple	5,945	0.2%
**Outpatient setting type^4^**		
Office	1,949,437	55.8%
Telehealth	220,068	6.3%
Emergency department	467,249	13.4%
Hospital-affiliated clinic	343,714	9.8%
Urgent care facility	275,245	7.9%
Health clinic	5,853	0.2%
Other	205,695	5.9%
Unknown	26,183	0.7%
**Number of visits per patient**		
1	807,974	23.1%
2 to 3	925,761	26.5%
4 to 5	526,159	15.1%
≥6	1,233,550	35.3%
**Outpatient setting borough^5^**		
Manhattan	1,143,104	32.7%
Bronx	545,502	15.6%
Brooklyn	920,876	26.4%
Queens	687,209	19.7%
Staten Island	196,753	5.6%
**Patient borough of residence^6^**		
Manhattan	562,158	16.1%
Bronx	589,253	16.9%
Brooklyn	971,477	27.8%
Queens	701,117	20.1%
Staten Island	194,918	5.6%
New York State (NYS)-Outside NYC	260,015	7.4%
Outside NYS	214,227	6.1%
Unknown	279	0.0%
**Visit diagnosis category** ^7^		
Asthma/Allergy	1,371,366	39.3%
Cough	659,655	18.9%
Viral upper respiratory tract infections	505,352	14.5%
Coronaviruses (including COVID-19)^8^	440,268	12.6%
Bronchitis/Bronchiolitis	126,716	3.6%
Influenza	74,245	2.1%
Non-suppurative Otitis media	43,728	1.3%
Multiple	272,114	7.8%
**Number of ARI diagnosis codes per Visit**		
1	3,111,779	89.1%
2 to 3	378,103	10.8%
≥4	3,562	0.1%
**Provider age at visit (years)**		
<35	249,180	7.1%
35–49	973,116	27.9%
50–64	1,105,410	31.6%
≥65	630,191	18.0%
Unknown	535,547	15.3%
**Provider type** ^9^		
Physician	2,952,077	84.5%
Nurse practitioner	336,605	9.6%
Physician assistant	190,955	5.5%
Other provider type	13,403	0.4%
Unknown	404	0.0%
**Physician specialty (Total N = 2,952,077 visits)** ^10^		
Internal medicine	879,354	29.8%
Internal medicine subspecialties	373,561	12.7%
Emergency medicine	285,041	9.7%
Family medicine	410,358	13.9%
Pediatric	598,708	20.3%
Allergy and Immunology	34,660	1.2%
Otolaryngology	78,114	2.7%
Other	292,281	9.9%
**Provider Gender ^2^**		
Male	1,768,915	50.6%
Female	1,724,529	49.4%

1 Visits were defined by a patient identification number, rendering provider identification number, and visit date.

2 Gender variables are listed from the health insurance claim, and it is unknown if they represent gender or sex at birth.

3 For payment type categories, third party payment includes but is not limited to private, employer, and liability insurance. Multiple payment types included Medicaid and Medicare, and Medicare or Medicaid and a third party insurance.

4 Outpatient settings were based on the Centers for Medicare and Medicaid Services definitions and were further categorized by the authors as shown in Supplemental Table 2.

5 Outpatient setting boroughs were based on county of provider mailing address.

6 Patient borough of residence was categorized based on patients’ 3-digit ZIP code.

7 *International Classification of Diseases, Tenth Revision, Clinical Modification* (*ICD-10-CM*) codes requested for diagnoses where an antibiotic is never indicated were categorized in Supplemental Table 1.

8 *ICD-10-CM* code U071 for COVID-19 was introduced March 18, 2020.

9 Provider types are categorized in Supplemental Table 4.

10 Physician provider specialties are categorized in Supplemental Table 5.

Asthma/allergy diagnoses were most common, assigned to >1.3 million visits (39.3%). Other frequent diagnoses were cough (18.9%), viral upper respiratory tract infections (14.5%), and coronaviruses (12.6%). Some visits (7.8%) were assigned codes from multiple diagnosis categories (eg, bronchitis/bronchiolitis and influenza) (Table 1).

Most ARI visits did not receive an inappropriate antibiotic prescription (94.9%). At least one inappropriate antibiotic prescription was made for 5.1% of visits, resulting in over 179,000 unnecessary antibiotic courses dispensed (Table 2). Inappropriate prescribing trended downwards from 6.9% in 2019 to 4.7% in 2022, with the lowest percentage occurring in 2021 (3.7%). The RR of inappropriate prescribing in 2022 relative to 2019 was 0.67 (95% CI: 0.66, 0.68) (Table 2).

**Table 2.** Unadjusted relative risk of an inappropriate antibiotic^1^ obtained by a patient that was prescribed at an outpatient visit^2^ for acute respiratory illnesses (ARI) by a New York City (NYC)-based provider, 2019–2022

	Total N	Visit received an inappropriate antibiotic prescription n (%)	Visit did not receive an inappropriate antibiotic prescription n (%)	Unadjusted relative risk using poisson regression with robust error variance (95% Confidence Interval)
**Total visits**	3,493,444	179,029 (5.1%)	3,314,415 (94.9%)	N/A
**Year of visit**				
2019	939,921	65,108 (6.9%)	874,813 (93.1%)	ref
2020	801,656	40,157 (5.0%)	761,499 (95.0%)	.73 (.72, .74)
2021	805,155	29,642 (3.7%)	775,513 (96.3%)	.54 (.54, .55)
2022	946,712	44,122 (4.7%)	902,590 (95.3%)	.67 (.66, .68)
**Patient age at visit (years) (N = 3,481,399)^3^**				
0–17	791,981	24,492 (3.1%)	767,489 (96.9%)	ref
18–24	175,654	8,302 (4.7%)	167,352 (95.3%)	1.59 (1.55, 1.63)
25–44	748,964	39,292 (5.2%)	709,672 (94.8%)	1.77 (1.74, 1.80)
45–64	974,773	61,021 (6.3%)	913,752 (93.7%)	2.15 (2.11, 2.18)
65–79	554,475	34,700 (6.3%)	519,775 (93.7%)	2.21 (2.17, 2.25)
80+	235,552	11,222 (4.8%)	224,330 (95.2%)	1.76 (1.72, 1.81)
**Patient gender (N = 3,482,585)** ^4,5^				
Male	1,427,035	69,193 (4.8%)	1,357,842 (95.2%)	ref
Female	2,055,550	109,836 (5.3%)	1,945,714 (94.7%)	1.11 (1.10, 1.12)
**Visit payment type (N = 3,493,443)** ^6,7^				
Third party	2,768,542	144,198 (5.2%)	2,624,344 (94.8%)	ref
Medicare (including Part D)	450,253	24,939 (5.5%)	425,314 (94.5%)	1.16 (1.14, 1.18)
Medicaid	268,604	9,577 (3.6%)	259,027 (96.4%)	.68 (.67, .70)
Multiple payment methods	5,945	309 (5.2%)	5,636 (94.8%)	1.05 (.95, 1.17)
Unknown	99	6 (6.1%)	93 (93.9%)	1.25 (.61, 2.55)
**Outpatient setting type^8^**				
Office	1,949,437	127,953 (6.6%)	1,821,484 (93.4%)	ref
Telehealth	220,068	11,194 (5.1%)	208,874 (94.9%)	.77 (.76, .79)
Emergency department	467,249	9,295 (2.0%)	457,954 (98.0%)	.30 (.29, .30)
Hospital-affiliated clinic	343,714	5,696 (1.7%)	338,018 (98.3%)	.25 (.24, .25)
Urgent care facility	275,245	23,156 (8.4%)	252,089 (91.6%)	1.25 (1.23, 1.27)
Health Clinic	5,853	191 (3.3%)	5,662 (96.7%)	.54 (.46, .62)
Other	205,695	1,142 (.6%)	204,553 (99.4%)	.10 (.10, .11)
Unknown	26,183	402 (1.5%)	25,781 (98.5%)	.28 (.26, .31)
**Number of visits per patient**				
1	807,974	48,063 (5.9%)	759,911 (94.1%)	ref
2 to 3	925,761	54,778 (5.9%)	870,983 (94.1%)	.98 (.97, .99)
4 to 5	526,159	28,564 (5.4%)	497,595 (94.6%)	.89 (.87, .90)
≥6	1,233,550	47,624 (3.9%)	1,185,926 (96.1%)	.65 (.64, .66)
**Outpatient setting borough ^9^**				
Manhattan	1,143,104	44,813 (3.9%)	1,098,291 (96.1%)	ref
Bronx	545,502	19,572 (3.6%)	525,930 (96.4%)	.95 (.93, .97)
Brooklyn	920,876	51,341 (5.6%)	869,535 (94.4%)	1.41 (1.39, 1.43)
Queens	687,209	46,270 (6.7%)	640,939 (93.3%)	1.72 (1.70, 1.74)
Staten Island	196,753	17,033 (8.7%)	179,720 (91.3%)	2.22 (2.17, 2.26)
**Patient borough of residence** ^10^				
Manhattan	562,158	22,136 (3.9%)	540,022 (96.1%)	ref
Bronx	589,253	18,911 (3.2%)	570,342 (96.8%)	.82 (.80, .84)
Brooklyn	971,477	50,426 (5.2%)	921,051 (94.8%)	1.30 (1.28, 1.32)
Queens	701,117	44,076 (6.3%)	657,041 (93.7%)	1.58 (1.55, 1.60)
Staten Island	194,918	16,995 (8.7%)	177,923 (91.3%)	2.23 (2.18, 2.27)
New York State (NYS)-Outside NYC	260,015	15,154 (5.8%)	244,861 (94.2%)	1.49 (1.46, 1.53)
Outside NYS	214,227	11,325 (5.3%)	202,902 (94.7%)	1.34 (1.31, 1.37)
Unknown	279	6 (2.2%)	273 (97.8%)	.69 (.36, 1.30)
**Visit diagnosis category** ^11^				
Asthma/Allergy	1,371,366	19,534 (1.4%)	1,351,832 (98.6%)	ref
Cough	659,655	36,318 (5.5%)	623,337 (94.5%)	3.88 (3.81, 3.96)
Viral upper respiratory tract infections	505,352	41,444 (8.2%)	463,908 (91.8%)	5.78 (5.67, 5.89)
Coronaviruses (including COVID-19)^12^	440,268	7,296 (1.7%)	432,972 (98.3%)	1.20 (1.17, 1.24)
Bronchitis/Bronchiolitis	126,716	32,370 (25.5%)	94,346 (74.5%)	18.03 (17.70, 18.38)
Influenza	74,245	1,900 (2.6%)	72,345 (97.4%)	1.79 (1.70, 1.88)
Non-suppurative Otitis media	43,728	6,886 (15.7%)	36,842 (84.3%)	11.62 (11.30, 11.95)
Multiple	272,114	33,281 (12.2%)	238,833 (87.8%)	8.50 (8.34, 8.66)
**Number of ARI diagnosis codes per Visit**				
1	3,111,779	143,732 (4.6%)	2,968,047 (95.4%)	ref
2 to 3	378,103	35,032 (9.3%)	343,071 (90.7%)	2.01 (1.99, 2.03)
≥4	3,562	265 (7.4%)	3,297 (92.6%)	1.93 (1.72, 2.15)
**Provider age at visit (years)**				
<35	249,180	6,811 (2.7%)	242,369 (97.3%)	ref
35–49	973,116	37,616 (3.9%)	935,500 (96.1%)	1.41 (1.37, 1.44)
50–64	1,105,410	68,008 (6.2%)	1,037,402 (93.8%)	2.25 (2.19, 2.31)
≥65	630,191	43,210 (6.9%)	586,981 (93.1%)	2.54 (2.48, 2.61)
Unknown	535,547	23,384 (4.4%)	512,163 (95.6%)	1.70 (1.65, 1.74)
**Provider Type** ^13^				
Physician	2,952,077	154,122 (5.2%)	2,797,955 (94.8%)	ref
Nurse practitioner	336,605	12,362 (3.7%)	324,243 (96.3%)	.76 (.75, .78)
Physician assistant	190,955	12,490 (6.5%)	178,465 (93.5%)	1.27 (1.25, 1.30)
Other provider type	13,403	52 (.4%)	13,351 (99.6%)	.12 (.09, .16)
Unknown	404	3 (.7%)	401 (99.3%)	.14 (.05, .44)
**Physician Specialty (N = 2,952,077)** ^14^				
Internal medicine	879,354	70,242 (8.0%)	809,112 (92.0%)	ref
Internal medicine subspecialties	373,561	17,119 (4.6%)	356,442 (95.4%)	.55 (.54, .56)
Emergency medicine	285,041	11,115 (3.9%)	273,926 (96.1%)	.46 (.45, .47)
Family medicine	410,358	29,262 (7.1%)	381,096 (92.9%)	.87 (.86, .88)
Pediatric	598,708	20,228 (3.4%)	578,480 (96.6%)	.39 (.38, .39)
Allergy and Immunology	34,660	209 (.6%)	34,451 (99.4%)	.09 (.08, .11)
Otolaryngology	78,114	3,075 (3.9%)	75,039 (96.1%)	.47 (.45, .49)
Other	292,281	2,872 (1.0%)	289,409 (99.0%)	.11 (.11, .12)
** Provider Gender^4^**				
Male	1,768,915	99,000 (5.6%)	1,669,915 (94.4%)	ref
Female	1,724,529	80,029 (4.6%)	1,644,500 (95.4%)	.83 (.82, .84)

1 Only new, oral antibiotics were included. For a complete list of antibiotics, see Supplemental Table 3.

2 Visits were defined by a patient identification number, rendering provider identification number, and visit date.

3 Excluded 12,045 visits, with patients of unknown age, none of which received an antibiotic prescription post-visit; inclusion prevented the model from converging.

4 Gender variables are listed from the health insurance claim, and it is unknown if they represent gender or sex at birth.

5 Excluded 10,859 visits, with patients of unknown gender, none of which received an antibiotic prescription post-visit; inclusion prevented the model from converging.

6 For payment type categories, third party payment includes but is not limited to private, employer, and liability insurance. Multiple payment types included Medicaid and Medicare, and Medicare or Medicaid and a third party insurance.

7 Excluded 1 visit with cash payment type, where an antibiotic prescription was not received post-visit; inclusion prevented the model from converging.

8 Outpatient settings were based on the Centers for Medicare and Medicaid Services definitions and were further categorized by the authors as shown in Supplemental Table 2.

9 Outpatient setting boroughs were based on county of provider mailing address.

10 Patient borough of residence was categorized based on patients’ 3-digit ZIP code.

11 *International Classification of Diseases, Tenth Revision, Clinical Modification* (*ICD-10-CM*) codes requested for diagnoses where an antibiotic is never indicated were categorized in Supplemental Table 1.

12 *ICD-10-CM* code U071 for COVID-19 was introduced March 18, 2020.

13 Provider types are categorized in Supplemental Table 4.

14 Physician provider specialties are categorized in Supplemental Table 5.

All adult patients had an increased risk for an inappropriate prescription relative to pediatric patients; the highest was among adults ages 65–79 (RR: 2.21; 95% CI: 2.17, 2.25). Relative to visits paid with third party insurance, the risk of an inappropriate prescription was higher for visits paid with Medicare (RR: 1.16; 95% CI 1.14, 1.18) and lower for visits paid with Medicaid (RR: 0.68; 95% CI: 0.67, 0.70) (Table 2).

Percentages of inappropriate prescribing were highest during visits in urgent care (8.4%), offices (6.6%), and telehealth (5.1%). Compared with office-based visits, urgent care visits were more likely to result in an inappropriate prescription (RR: 1.25; 95% CI:1.23, 1.27). By borough, inappropriate prescribing was highest in Staten Island (8.7%) and Queens (6.7%), with risk of an inappropriate prescription in Staten Island more than twice as high relative to visits in Manhattan (RR: 2.22; 95% CI: 2.17, 2.26) (Table 2).

The lowest percentages of inappropriate prescribing were for visits with asthma/allergy (1.4%), coronaviruses (1.7%), and influenza (2.6%) diagnoses. Visits for bronchitis/bronchiolitis, non-suppurative otitis media, and viral upper respiratory tract infections had the highest percentages of inappropriate prescribing and high RR for an antibiotic prescription relative to asthma/allergy diagnoses: 25.5% (RR: 18.03; 95% CI: 17.70, 18.38), 15.7% (RR: 11.62; 95% CI: 11.30, 11.95), and 8.2% (RR: 5.78; 95% CI: 5.67, 5.89), respectively. Visits assigned codes from >1 diagnosis category were more likely to be associated with an inappropriate prescription compared with those assigned one code (RR: 2.01; 95% CI 1.99, 2.03 for 2–3 codes and RR: 1.93; 95% CI: 1.72, 2.15 for ≥4 codes) (Table 2). The most common antibiotic class inappropriately prescribed across all visits was macrolides, with variation across diagnosis categories (Supplemental Table 6).

The RR of inappropriate antibiotic prescribing increased with provider age; providers aged ≥ 65 years had a RR of 2.54 (95% CI: 2.48, 2.61) relative to providers aged < 35 years. By provider type, PAs had the highest percentage of inappropriate prescribing (6.5%) followed by physicians (5.2%); risk of inappropriate prescribing for PAs relative to physicians was 1.27 (95% CI: 1.25, 1.30). By physician specialty, the highest percentage of inappropriate prescribing was among internal medicine physicians (8.0%) and family medicine physicians (7.1%). Relative to visits with internal medicine physicians, the risk of inappropriate prescribing from all other physician specialties was lower. Risk of inappropriate prescribing by emergency medicine physicians was less than half of that relative to internal medicine physicians (RR: 0.46; 95% CI: 0.45, 0.47) (Table 2).

## Discussion

Inappropriate antibiotic prescribing occurred in approximately 5% of ARI visits in NYC. It was highest for bronchitis/bronchiolitis and non-suppurative otitis media diagnoses; visits in office settings and setting types designed for efficiency such as urgent care and telehealth; visits with older patients or older providers; with internal medicine physicians, family medicine physicians, and PA providers. Geographical differences in inappropriate prescribing were also noted with high percentages in certain areas of NYC, including Staten Island and Queens. While inappropriate prescribing decreased from 2019 (6.9%) to 2022 (4.7%), the impact of the COVID-19 pandemic on this trend could not be adequately evaluated with the data available. Decreases in inappropriate prescribing during the pandemic’s height were seen nationally, but investigations have noted recent increases, trending toward prepandemic levels.^22^ Our data may reflect similar trends with inappropriate prescribing rising from a nadir of 3.7% in 2021 to 4.7% in 2022.

Though inappropriate prescribing in NYC is lower than findings reported nationally or in other regions,^8^ the results from this analysis are consistent with literature to date which indicates more appropriate or overall, less antibiotic prescribing in New York State (NYS) and NYC than reported elsewhere. Regional differences in antibiotic prescribing have been noted nationally, with prescribers in the northeast generally showing less inappropriate prescribing for ARI than prescribers in the South or Midwest.^9^ Even throughout NYS, differences in antibiotic prescribing have been noted, with NYC generally having less overall prescribing than other NYS counties.^23^ The overlapping period of assessment with the COVID-19 pandemic could also have contributed to the lower percentages of inappropriate prescribing seen in this study compared to those previously reported. Notably, however, one study found that even within a single healthcare enterprise, there was great variability in prescribing regionally. Prior to AS interventions, inappropriate prescribing at visits for similar respiratory illnesses throughout the Mayo Clinic healthcare enterprise in three states during January 1, 2019 to June 30, 2020, ranged from 7 to 27%.^24^ Despite overall differences in inappropriate prescribing, the associated factors were similar to those reported elsewhere, such as the diagnoses and setting types.^8,24^

Inappropriate prescribing was highest for visits with diagnosis of bronchitis/bronchiolitis (25.5%) or non-suppurative otitis media (15.7%) while influenza (2.6%) and coronavirus (including COVID-19) (1.7%) visits had among the lowest percentages. Though information about point-of-care testing was unavailable, it is notable that conditions for which none currently exist had higher percentages of inappropriate prescribing^10,25^ since diagnostic uncertainty is one factor that can influence inappropriate antibiotic prescribing among clinicians.^12,26,27^ Investment in point-of-care testing, alongside provider audit and feedback and patient education may contribute to the multi-pronged approach to evidence-based AS interventions.

Visits in urgent care (8.4%) and telehealth (5.1%) were among the highest settings with inappropriate prescribing, as reported elsewhere.^9,10,24,28,29^ Urgent care and telehealth settings are gaining traction due to convenience, expanded access, and lower costs.^24,30–32^ Given their growing popularity and the relatively high percentages of inappropriate prescribing identified, focusing stewardship efforts in these settings could be impactful. Drivers of inappropriate prescribing relevant to these settings include time constraints during visits and a lack of established patient-provider relationships.^26^ Promising AS initiatives in urgent care should be considered, such as patient and provider education or data tools to guide prescribing embedded in dashboards or electronic health record systems.^7,24,33^ Inappropriate prescribing was also elevated at office visits (6.6%); given their frequency for ARI visits (55%), offices may also represent an ideal setting for AS initiatives.

The highest percentages of inappropriate prescribing by physician specialties and provider types were found among internal medicine physicians (8.0%), family medicine physicians (7.1%), and PAs (6.5%). These specialties and providers have ranked as the highest inappropriate prescribers elsewhere.^9^ Conversely, inappropriate prescribing among NPs in this study was relatively low (3.7%), highlighting the need to disaggregate NPs and PAs for evaluation of prescribing practices. Providing feedback on prescribing practices, which can include comparison to peers in the same specialty along with educational resources, is one strategy to engage providers to improve antibiotic prescribing.^1^ Like findings reported elsewhere, increased inappropriate prescribing also occurred among older providers, specifically providers ages 50–64 years (6.2%) and ≥65 years (6.9%).^9,24^ It has been suggested that older providers may rely more on clinical experience rather than engaging with current antibiotic use best practices.^26^ Requiring continuing education that incorporates AS principles may help reduce this gap.

Conversely, certain setting, patient, and provider characteristics were associated with low percentages of inappropriate prescribing such as pediatric physician specialties (3.4%) and at visits with pediatric patients (3.1%). More judicious prescribing in pediatric populations has been noted nationally and might be related to tailored AS initiatives in pediatric settings.^6,8,10,34^ Moreover, percentages of inappropriate prescribing were low among emergency medicine physician specialties (3.9%) and in emergency departments (2.0%), as previously reported.^9,24^ Various explanations have been proposed for the higher quality of antibiotic prescribing in emergency departments including potential positive impacts from AS initiatives or prescribing metrics already established in their respective hospitals.^30,35^

Various considerations are needed when designing AS initiatives and considering both absolute and relative numbers of inappropriate prescriptions can be important. For example, implementing interventions in urgent care could be justified by the high percentage of inappropriate prescribing identified there. However, offices had the second highest percentage of inappropriate prescribing and contributed the most inappropriate prescriptions among setting types, magnitudes higher than urgent care, highlighting an important opportunity. Other important considerations include assessing what resources are available to be allocated and whether large- or small-scale interventions are feasible. Overall, the most impactful places to focus interventions or future research are likely where both percentages of inappropriate prescribing and the absolute contribution of inappropriate prescriptions are highest, such as among internal medicine physicians.

Further study is needed to uncover the drivers behind observed differences in inappropriate prescribing. For example, the percentage of inappropriate prescribing was over 4-fold higher in urgent care (8.4%) than in emergency departments (2.0%), but both settings receive acutely ill patients. Comparing these settings by their patient populations, provider types, available diagnostic tests, and access to AS resources would be useful to better understand how to tailor interventions.

Study limitations may have underestimated inappropriate prescribing. The analysis would not capture a prescription if the provider listed on the prescription differed from the visit provider. Prescriptions written during a visit but not retrieved from the pharmacy were unavailable for inclusion. Using dispensed antibiotics as a proxy for prescriptions is likely to underestimate inappropriate prescribing. However, this limitation could not be evaluated. There are considerations for why prescriptions may remain unfilled. This limitation may miss inappropriate prescribing trends for certain groups. For example, individuals with low income may be both less likely to receive and fill prescriptions.^36,37^ Therefore, it is unclear if inappropriate prescribing may be under- or over- estimated when only dispensed antibiotics could be assessed. As noted earlier, the study period overlapping with the COVID-19 pandemic’s height limited interpretation of overall lower percentages of inappropriate prescribing identified. However, overestimation was also possible. Using *ICD-10-CM* codes as a proxy for diagnosis without clinical information, a method used to assess widespread patterns, may not reflect the full picture of the patient’s condition.^9,10,20^ Overestimation is especially important to consider for patients with multiple visits. Because no variable could link prescriptions to the specific visit at which they were prescribed and prescriptions were instead matched based on patient, provider, and visit information, a prescription could have been ascribed to more than one visit or to the incorrect visit. As only outpatient prescribing was assessed, these results cannot be generalized to patients or providers in hospitals, nursing homes, or long-term care facilities. To increase validity, the data received restricted visits to antibiotic-inappropriate diagnoses only, excluding visits with concurrent diagnoses where an antibiotic could be appropriate. While using a short, 3-day time frame could have resulted in underestimation, it decreased the likelihood of ascribing an antibiotic to the wrong visit and drastically overestimating inappropriate prescribing. This analysis is further strengthened by the broad coverage of IQVIA data. These data allowed for assessment across a wide patient age distribution, public and privately insured patients, and across a variety of outpatient setting types.

## Conclusions

This study highlighted opportunities for targeted interventions to improve prescribing. Visit-level factors associated with inappropriate prescribing were identified, potentially rooted in diagnostic uncertainty, a lack of established patient-provider relationships, or reliance on clinical experience rather than updated guidance. While these findings can help prioritize limited stewardship resources, such as tailoring interventions in low-performing setting types or geographic areas, future study should be directed at clarifying the main drivers of prescribing pattern disparities in areas with increased inappropriate prescribing. Intersectional interventions that target not only the setting type, but also focus on the provider and diagnosis, may have the greatest impact on reducing inappropriate antibiotic prescribing.

## Supporting information

10.1017/ash.2026.10351.sm001 Santiago et al. supplementary material Santiago et al. supplementary material

## Data Availability

The datasets from IQVIA analyzed during the current study are proprietary and are not publicly available.

## Supplementary material

The supplementary material for this article can be found at https://doi.org/10.1017/ash.2026.10351.
