# Supplementary material for: Inappropriate antibiotic prescribing for acute respiratory illnesses in outpatient settings in New York City, 2019–2022
Source: Antimicrob Steward Healthc Epidemiol. 2026 Apr 20;6(1):e116. doi: 10.1017/ash.2026.10351 (PMC13104526; doi:10.1017/ash.2026.10351)
Supplement: Santiago et al. supplementary material [file S2732494X26103519sup001.pdf]

Supplemental Materials

Supplemental Figure 1: Flow Diagram of Provider Exclusion for the Analysis of Inappropriate Antibiotic Prescribing at Outpatient Visits for Acute Respiratory Illnesses in New York City

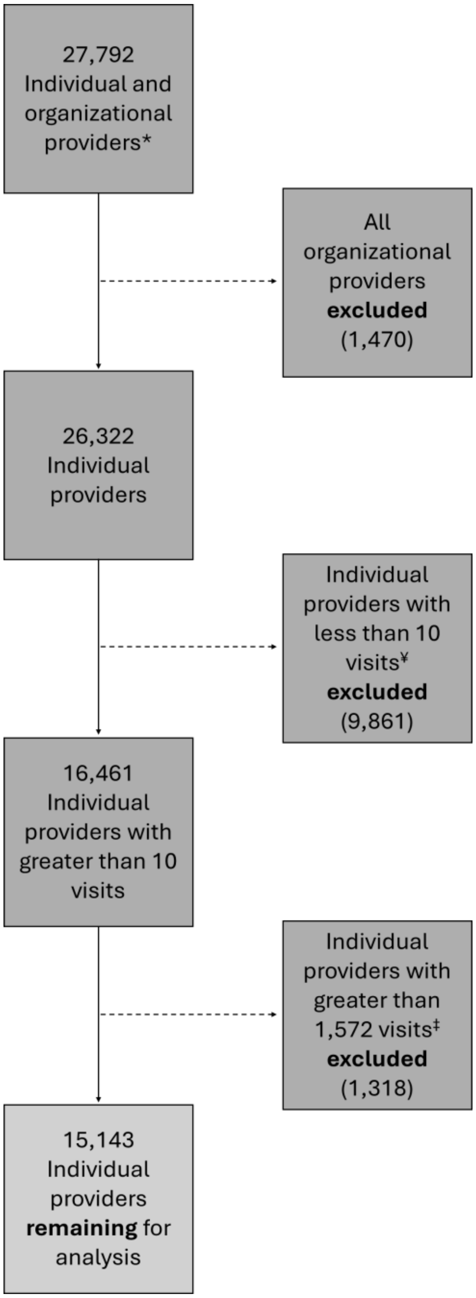

*National Provider Identification Number (NPI) identifies providers as individual or organizational. Only providers with an individual NPI designation were retained in the analytic dataset.
¥Consistent with previous methodology, providers with <10 antibiotic-inappropriate acute respiratory illness visits during the study period were excluded for stability (King et al., 2023).
‡Providers identified with total visits in excess of an extreme upper outlier were excluded to improve validity of provider visit volume.

**Supplemental Table 1:** ***International Classification of Diseases, Tenth Revision, Clinical Modification*** **(*ICD-10-CM)*** **Diagnosis Codes for Antibiotic-Inappropriate Acute Respiratory Illnesses**	
**Suitable Diagnosis Category[1]**	**Data Requested from IQVIA[2]**
Non-suppurative Otitis media	H6500, H6501, H6502, H6503, H6504, H6505, H6506, H6507, H65111, H65112, H65113, H65114, H65115, H65116, H65117, H65119, H65191, H65192, H65193, H65194, H65195, H65196, H65197, H65199, H6520, H6521, H6522, H6523, H6530, H6531, H6532, H6533, H65411, H65412, H65413, H65419, H65491, H65492, H65493, H65499, H6590, H6591, H6592, H6593, H671, H672, H673, H679
Cough	R05, R051, R052, R053, R054, R055, R056, R058, R059
Viral upper respiratory tract infections	J00, J040, J0410, J0411, J042, J0430, J0431, J050, J0510, J0511, J060, J069
Influenza	J09X1, J09X2, J09X3, J09X9, J1000, J1001, J1008, J101, J102, J1081, J1082, J1083, J1089, J1100, J1108, J111, J112, J1181, J1182, J1183, J1189
Viral Pneumonia[3]	J120, J121, J122, J123, J1289, J129
Bronchitis/Bronchiolitis	J200, J201, J202, J203, J204, J205, J206, J207, J208, J209, J210, J211, J218, J219, J22, J40
Asthma/Allergy	J300, J301, J302, J305, J3081, J3089, J309, J310, J311, J312, J4520, J4521, J4522, J4530, J4531, J4532, J4540, J4541, J4542, J4550, J4551, J4552, J45901, J45902, J45909, J45990, J45991, J45998, T7840XA, T7840XD, T7840XS, T7841XA, T7841XD, T7841XS, T7849XA, T7849XD, T7849XS
Coronavirus (including COVID-19)[4]	U071, J1281, J1282
Multiple	This was not a categorization in the IQVIA dataset, but was assigned by the authors to visits with two or more *ICD-10-CM* codes that spanned more than one diagnosis classification category

[1]These categories were created by the authors with reference to previous categorizations to organize related *International Classification of Diseases, Tenth Revision, Clinical Modification ICD-10-CM* codes (King et al., 2023; Young et al., 2020).
[2]Not all *ICD-10-CM* codes requested were received in the final dataset. Any that were received were categorized into these respective classifications.
[3]Of note, no diagnosis codes for viral pneumonia were received in the analytic dataset and therefore, this category does not appear in the data tables.
[4]All codes for coronavirus were grouped together since the ICD-10 code U071 was not introduced until March 18, 2020, and providers may have been using other codes prior to this.

Supplemental Table 2: Outpatient Setting Location Categorizations[1]	
**Category**	**Data values comprising the category**
Office	Office
Telehealth	Telehealth provided in patient's home, Telehealth provided other than in patient's home
Emergency Department	Emergency room - hospital
Outpatient hospital clinic	Off campus-outpatient hospital, On campus-outpatient hospital
Urgent care facility	Urgent care facility
Health Clinic	Independent clinic, Public health clinic, Rural health clinic, Walk-in retail health clinic
Other	Ambulance - land, Ambulatory surgical center, Birthing center, Comprehensive outpatient rehabilitation facility, Federally qualified health center, Hospice, Independent laboratory, Military treatment facility, Mobile unit, Non-residential opioid treatment facility, Other place of service, Pharmacy, Psychiatric facility-partial hospitalization, School, Tribal 638 provider-based facility
Unknown	Two or more outpatient setting categories were reported for the defined visit and it is unclear where the visit took place

[1] Outpatient setting categories were based on the Centers for Medicare & Medicaid Services definitions (Centers for Medicare & Medicaid Services, 2024).

Supplemental Table 3: Antibiotic Class Categorizations	
**Category**	**Data values comprising the category**
Cephalosporins	Cefaclor, Cefaclor er, Cefadroxil, Cefdinir, Cefixime, Cefpodoxime proxetil, Cefprozil, Cefuroxime axetil, Cephalexin, Suprax
Fluoroquinolones	Cipro, Ciprofloxacin hcl, Ciprofloxacin hydrochlori, Levofloxacin, Moxifloxacin hydrochlorid, Ofloxacin
Macrolides	Azithromycin, Clarithromycin, Clarithromycin er, Dificid, Erythromycin, Erythromycin base, Erythromycin dr, Erythromycin ethylsuccina, Zithromax, Zithromax tri-pak, Zithromax z-pak
Penicillins	Amoxicillin, Amoxicillin/clavulanate p, Ampicillin, Augmentin, Dicloxacillin sodium, Penicillin V potassium
Other Antibiotic Class	Bactrim ds, Dapsone, Sulfamethoxazole/trimetho, Sulfatrim pediatric, Atovaquone, Avidoxy, Clindamycin hcl, Clindamycin hydrochloride, Clindamycin palmitate hcl, Doxycycline, Doxycycline hyclate, Doxycycline hyclate dr, Doxycycline monohydrate, Fosfomycin tromethamine, Linezolid, Metronidazole, Minocycline hcl, Minocycline hydrochloride, Monurol, Neomycin sulfate, Nitrofurantoin macrocryst, Nitrofurantoin monohydrat, Solosec, Tetracycline hydrochlorid, Tinidazole, Trimethoprim, Vancomycin hydrochloride, Xifaxan

Supplemental Table 4: Provider Type Categorizations	
**Category**	**Data values comprising the category**
Physician	See Supplemental Table 5 for physician specialty categorizations where all physician categories are included
Nurse practitioner	Nurse practitioner
Physician assistant	Physician assistant
Other provider type	Acupuncturist, Advanced registered nurse, Ancillary services, Behavioral health & social services, Certified nurse anesthetist, Clinical social worker, Licensed practical nurse, Nurse midwife, Nutritionist, Occupational therapy, Occupational therapy - assistant, Optometrist, Registered dietician, Registered nurse, Student, Health care, Technologist - audiology/speech/language
Unknown	Not applicable, Unspecified

Supplemental Table 5: Physician Specialty Categorizations	
**Category**	**Data values comprising the category**
Internal Medicine	Internal medicine, Geriatric medicine (internal medicine), Internal medicine/pediatrics, Internal medicine/psychiatry, Internal medicine/preventive medicine
Internal Medicine Subspecialties	Pulmonary disease, Cardiovascular disease, Pulmonary critical care medicine, Gastroenterology, Infectious disease, Nephrology, Hematology/oncology, Endocrinology, Diabetes & metabolism, Rheumatology, Critical care medicine (internal medicine), Hospitalist, Interventional cardiology, Hematology (internal medicine), Medical oncology, Hospice & palliative medicine, Sleep medicine, Clinical cardiac electrophysiology, Addiction medicine, Hospice & palliative medicine (internal medicine), Reproductive endocrinology & infertility, Advanced heart failure & transplant cardiology, Nuclear cardiology, Clinical informatics (internal medicine), Sports medicine (internal medicine), Palliative medicine, Hepatology, Transplant hepatology (internal medicine)
Emergency Medicine	Emergency medicine, Internal medicine/emergency medicine, Emergency medical services, Medical toxicology (emergency medicine), Sports medicine (emergency medicine)
Family Medicine	Family medicine, Sports medicine (family medicine), Internal medicine/family medicine, Geriatric medicine (family medicine)
Pediatric	Pediatrics, Pediatric emergency medicine (pediatrics), Pediatric emergency medicine, Pediatric pulmonology, Pediatric radiology, Adolescent medicine (pediatrics), Pediatric allergy, Pediatric nephrology, Pediatric cardiology, Pediatric endocrinology, Pediatric infectious disease, Pediatric hematology/oncology, Pediatric otolaryngology, Pediatric critical care medicine, Child neurology, Pediatric anesthesiology, Pediatric gastroenterology, Sports medicine (pediatrics), Pediatrics/emergency medicine, Pediatric rheumatology, Developmental/behavioral pediatrics, Child & adolescent psychiatry, Pediatrics/psychiatry/child & adolescent psychiatry, Pediatric surgery, Pediatric hospital medicine (pediatrics)
Allergy & Immunology	Allergy & immunology, Immunology, Allergy
Otolaryngology	Otolaryngology, Head & neck surgery, Neurotology (otolaryngology), Plastic surgery within the head & neck (otolaryngology)

Other	Diagnostic radiology, Neuroradiology, Obstetrics & gynecology, Radiology, General surgery, General practice, Anesthesiology, Physical medicine & rehabilitation, Vascular & interventional radiology, Neurology, General preventive medicine, Physical therapy, Nuclear medicine, Ophthalmology, Occupational medicine, Audiology, Abdominal radiology, Anatomic/clinical pathology, Psychiatry, Musculoskeletal radiology, Urology, Dermatology, Orthopedic surgery, Neonatal-perinatal medicine, Vascular surgery, Pain medicine, Thoracic surgery, Surgical critical care (surgery), Maternal & fetal medicine, Hematology (pathology), Nuclear radiology, Nutrition, Sports medicine (orthopedic surgery), Podiatrist, Critical care medicine (anesthesiology), Gynecology, Sports medicine (physical medicine & rehabilitation), Pharmacist, Clinical pathology, Public health & general preventive medicine, Psychology, Trauma surgery, Neurological surgery, Selective pathology, Plastic surgery, Other specialty, Medical genetics, Regional anesthesiology and acute pain medicine (anesthesiology), Clinical neurophysiology, Adult cardiothoracic anesthesiology, Psychiatry/neurology, Pain medicine (anesthesiology), Pain medicine (physical medicine & rehabilitation), Dermatopathology, Forensic psychiatry, Neuromuscular medicine (neurology), Neuromuscular medicine (physical medicine & rehabilitation), Cytopathology, Clinical pharmacology, Colon & rectal surgery, Abdominal surgery, Transplant surgery, Facial plastic surgery, Gynecological oncology, Interventional radiology-integrated, Oral & maxillofacial surgery, Vascular medicine, Radiation oncology, Addiction psychiatry, Musculoskeletal oncology, Chiropractic, Neuropathology, Hand surgery, Surgical oncology, Blood banking/transfusion medicine, Osteopathic manipulative medicine, Female pelvic medicine & reconstructive surgery, Geriatric psychiatry, Anatomic pathology

Supplemental Material 1, Example Code

Sample code linking IQVIA's Medical Claims (Dx) and Longitudinal Prescription Claims (LRx) datasets:

```
proc sql;
create table link_data as
select A.*,  B.*
from dx as A
left join rx as B
on A.patient_id = B.patient_id
and A.rendering_provider_id = B.prescriber_id
and 0 <= B.prescription_date- A.visit_date <= 3;
quit;
```

Example Poisson regression with robust standard error variance code for provider gender:

```
proc genmod data=RxDx_data_ind;
class rendering_provider_id patient_id provider_gender (ref='M') /param=reference ref=first;
model script= provider_gender / dist=poisson link=log;
repeated subject=rendering_provider_id*patient_id/ type=cs;
estimate 'Provider Gender Female' provider_gender 1 /exp;
run;
```

- Class statement:
  - Include any independent variables that will be included in the model (i.e. provider_gender) as well as any variables that are part of the correlation structure. In variables that are part of the correlation structure are the variables that violate the assumption of independence that will be included in the repeated subject statement (i.e. rendering_provider_id, patient_id).
  - In the (**ref=**) option the reference category selected should be indicated (in this example male designated M).*
  - The **param=** option specifies the parameterization for the variable(s) in class. Param=ref specifies reference cell coding and ref=first specifies the first ordered level as a reference.
- Model Statement:
  - The structure of this statement is the dependent variable= independent variable(s). The **dist=** is where the model being run is specified(in this case Poisson since the model used in this study was a Poisson regression). Poisson regression allows for modeling of data that is not normally distributed. The **link=** option appropriate is where the link function is specified for the model. Link=log is commonly used for Poisson regression and when modeling relative risks.
- Repeated subject statement:
  - This statement is used to specify the covariance structure, where **type=** is used to specify the working correlation matrix structure. Type=cs specifies that the correlation

matrix structure is exchangeable which is appropriate when the time between measurements does not influence the relationship between the correlated variables that violate the assumption of independence.

- Estimate statement:
  - This statement prints output for the model and the **exp=** option includes standard error and confidence limits for the association.

*In this study, reference categories were selected based on the largest category, the earliest period or youngest age group, or aimed for consistency across variables with similar categories.

Supplemental Table 6: Outpatient Visit[1] Acute Respiratory Illness Diagnoses Category[2] by the Applicable Inappropriate Antibiotic[3] Prescribed by a New York City Provider, 2019-2022 (N=3,493,444)

	**Non-suppurative Otitis Media**	**Viral Upper Respiratory Tract Infections**	**Influenza**	**Cough**	**Bronchitis/ Bronchiolitis**	**Asthma/ Allergy**	**Coronaviruses (including COVID-19)[4]**	**Multiple Diagnosis Categories**	***Total Visits with Antibiotic Class Prescribed[3]***
	N (%)	N (%)	N (%)	N (%)	N (%)	N (%)	N (%)	N (%)	N
**Antibiotic Class**									
Cephalosporins	685 (1.6%)	1,686 (0.3%)	135 (0.2%)	1,476 (0.2%)	1,639 (1.3%)	1,287 (0.1%)	209 (0.0%)	1,770 (0.7%)	8,887 (0.3%)
Fluoroquinolones	133 (0.3%)	1,686 (0.3%)	86 (0.1%)	2,155 (0.3%)	2,915 (2.3%)	1,750 (0.1%)	305 (0.1%)	2,055 (0.8%)	11,085 (0.3%)
Macrolides	683 (1.6%)	25,819 (5.1%)	966 (1.3%)	22,695 (3.4%)	20,416 (16.1%)	8,850 (0.6%)	5,224 (1.2%)	20,761 (7.6%)	105,414 (3.0%)
Penicillins	5,220 (11.9%)	10,554 (2.1%)	593 (0.8%)	5,607 (0.8%)	5,326 (4.2%)	4,297 (0.3%)	598 (0.1%)	6,524 (2.4%)	38,719 (1.1%)
Other[5]	165 (0.4%)	1,699 (0.3%)	120 (0.2%)	4,385 (0.7%)	2,074 (1.6%)	3,350 (0.2%)	960 (0.2%)	2,171 (0.8%)	14,924 (0.4%)
None Prescribed	36,842 (84.3%)	463,908 (91.8%)	72,345 (97.4%)	623,337 (94.5%)	94,346 (74.5%)	1,351,832 (98.6%)	432,972 (98.3%)	238,833 (87.8%)	3,314,415 (94.9%)
Total Visits with Given Diagnosis	43,728	505,352	74,245	659,655	126,716	1,371,366	440,268	272,114	3,493,444

[1]Visits were defined by a patient identification number, rendering provider identification number, and service date.
[2]Categorized using *International Classification of Diseases, Tenth Revision, Clinical Modification* (*ICD-10-CM*) codes for these diagnoses where an antibiotic is never indicated. Categorization of *ICD-10-CM* codes into each of the groupings here is available in Supplemental Table 1.
[3]These refer to a prescribed antibiotic that was subsequently picked up at a pharmacy by a patient. Only new, oral antibiotics were included.
[4]*ICD-10-CM* code U071 for COVID-19 was introduced March 18, 2020.
[5]Other includes other antibiotics or multiple classes prescribed at a single visit.

Centers for Medicare & Medicaid Services. (2024, May 2). *Place of Service Code Set*. https://www.cms.gov/medicare/coding-billing/place-of-service-codes/code-sets

King, L. M., Kusnetsov, M., Filippoupolitis, A., Arik, D., Bartoces, M., Roberts, R. M., Tsay, S. V., Kabbani, S., Bizune, D., Rathore, A. S., Valkova, S., Eleftherohorinou, H., & Hicks, L. A. (2023). Using machine learning to examine drivers of inappropriate outpatient antibiotic prescribing in acute respiratory illnesses. *Infect Control Hosp Epidemiol*, *44*(5), 786-790. https://doi.org/10.1017/ice.2021.476

Young, E. H., Panchal, R. M., Yap, A. G., & Reveles, K. R. (2020). National Trends in Oral Antibiotic Prescribing in United States Physician Offices from 2009 to 2016. *Pharmacotherapy*, *40*(10), 1012-1021. https://doi.org/10.1002/phar.2456
